# Anterior cervical corpectomy and fusion for blastomycosis causing destruction of C6 vertebra: a case report

**DOI:** 10.1186/s13256-015-0762-x

**Published:** 2015-11-25

**Authors:** Kushal R. Patel, Michal Szczodry, Sergey Neckrysh, Krzysztof Siemionow

**Affiliations:** Department of Orthopedic Surgery, University of Illinois Hospital & Health Sciences System, 835 S Wolcott Avenue, Chicago, IL 60612 USA

**Keywords:** Blastomycosis, Cervical spine, Corpectomy, Fungal infection, Osteomyelitis

## Abstract

**Introduction:**

We describe a patient who had cervical spine osteomyelitis caused by *Blastomyces dermatitidis* that resulted in cord compression and cervical spine instability.

**Case presentation:**

A 25-year-old Hispanic woman presented with fever, sweats, neck pain, and an enlarging neck mass with purulent discharge after sustaining a C6 vertebral body fracture. Magnetic resonance imaging confirmed C6 vertebral osteomyelitis, demonstrated by vertebral body destruction, cervical spine instability, prevertebral abscess, and spinal cord compression. She underwent C6 anterior cervical corpectomy and fusion, with fungal cultures confirming *Blastomyces dermatitidis.*

**Conclusions:**

Anterior cervical corpectomy and fusion successful debrided, decompressed, and restored cervical spine stability in a patient with vertebral osteomyelitis caused by *Blastomyces dermatitidis.* The patient was subsequently treated with a 1-year course of itraconazole and had no recurrence of infection 4 years postoperatively.

## Introduction

*Blastomyces dermatitidis* is a thermally dimorphic fungus endemic to North America [1]. The most common primary site of infection associated with *B. dermatitidis* is pneumonia with extrapulmonary dissemination occurring in 25 to 50 % of diagnosed cases, usually via the lymphohematogenous route [1, 2]. Osteomyelitis has been found in about 25 % of cases with extrapulmonary manifestations [3]. In 75 % of patients with osseous blastomycosis, pulmonary disease is also found at time of presentation [4]. We report a case of cervical spine osteomyelitis caused by *B. dermatitidis* resulting in C6 vertebral body destruction and subsequent cervical spine instability. The patient was successfully treated with anterior cervical corpectomy and fusion (ACCF) and itraconazole.

## Case presentation

A 25-year-old immunocompetent Hispanic woman sustained a mechanical fall after slipping on ice and hitting her chin on the ground. Two weeks after the injury, she began to develop neck pain that was not present immediately after the injury. She presented to an emergency department and X-rays of her cervical spine were obtained. The X-rays were found to be normal and she was discharged with pain medication. After another 2 weeks of persistent, non-improving neck pain, she returned to the emergency department of the referring hospital for evaluation. Cervical spine X-rays, cervical spine computed tomography (CT) scan without contrast, and a cervical spine magnetic resonance imaging (MRI) with and without contrast were obtained. CT and MRI revealed a right-sided paraspinal soft tissue mass and an osteolytic lesion of the C6 vertebral body, causing a compression deformity. Given the imaging findings, she was admitted for further diagnostic testing to evaluate for possible malignancy.

A chest X-ray and a chest CT scan with contrast performed during her hospitalization revealed a 3.6×1.5 cm mass in the middle lobe of her right lung. She additionally underwent an ultrasound-guided biopsy of the mass in her neck and a CT-guided biopsy of the mass in the middle lobe of her right lung. The biopsy of her neck mass was negative for malignancy but revealed multinucleated histiocytes, neutrophils, and inflammatory cells. The specimen was sent for Gram stain, acid-fast bacilli (AFB)/fungal smear, and cultures: aerobic/anaerobic, fungal, and tuberculosis (TB); all of which were negative. The biopsy of the lung mass was deemed inadequate. The patient failed to follow-up in clinic for biopsy results and was lost to follow-up.

**Fig. 1:**
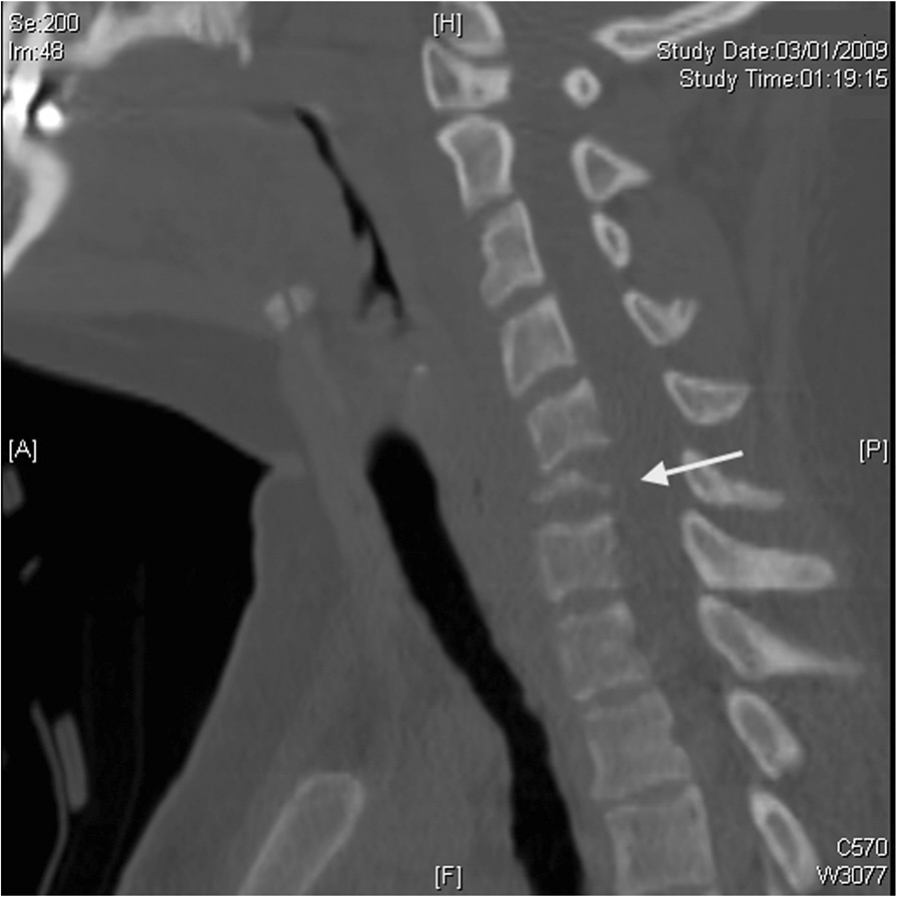
Preoperative sagittal computed tomography showing near complete destruction of C6 vertebral body (arrow)

**Fig. 2:**
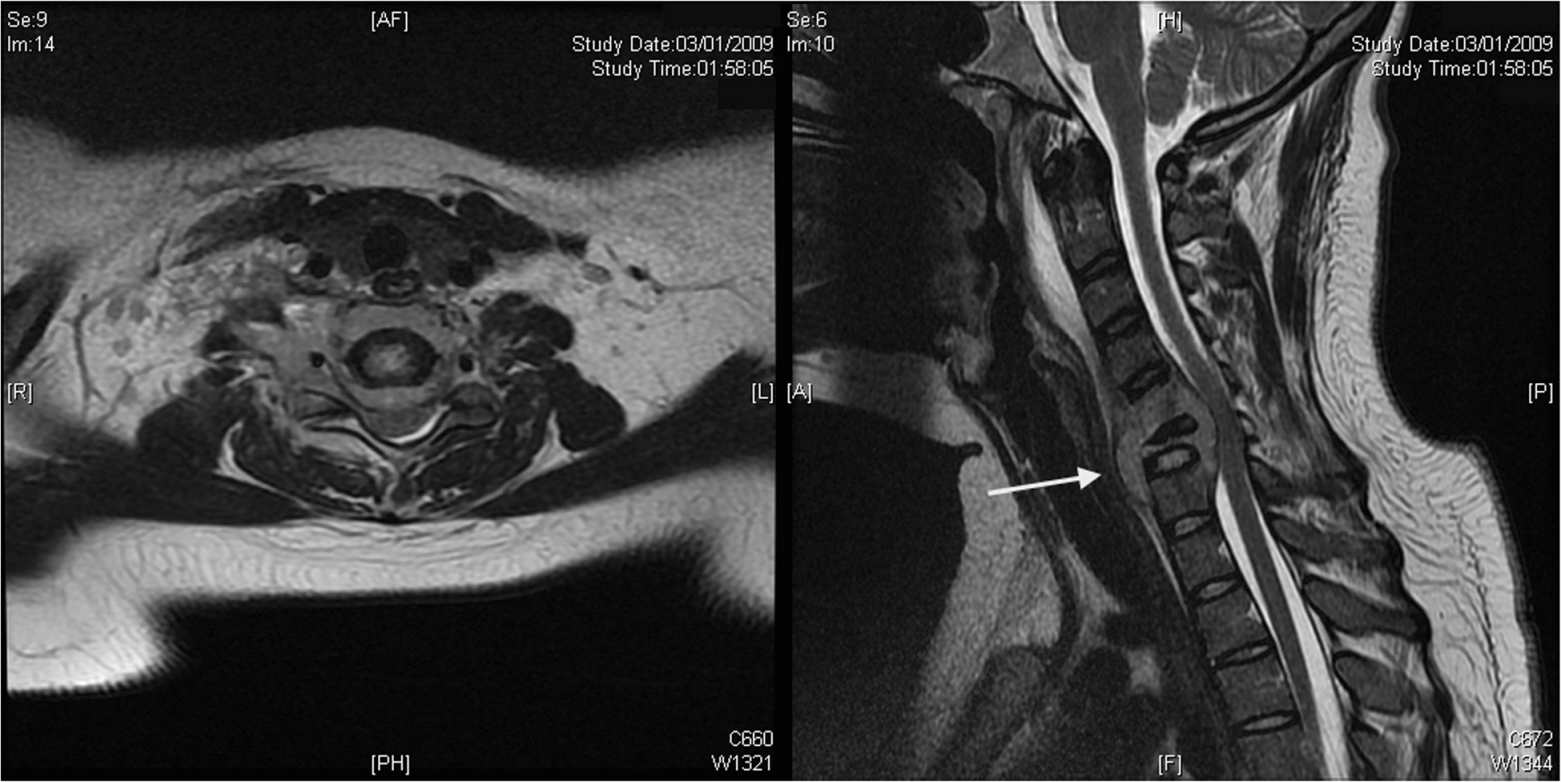
Preoperative sagittal (*right*) and axial at the C6 level (*left*) T2-weighted fast spin-echo magnetic resonance imaging views. The views show C6 osteomyelitis with significant vertebral body destruction (arrow), vertebral abscess at C6 level with posterior subligamentous extension into the spinal canal including mild posterior displacement and compression of cervical spinal cord

Four weeks later, she returned to the referring hospital’s emergency department complaining of a week of fevers, sweats, neck pain, as well as an enlarging neck mass with purulent discharge from the biopsy site. Repeat CT of the cervical spine and MRI of the cervical spine with and without contrast were obtained. Imaging revealed C6 osteomyelitis with significant vertebral body destruction and possible C5 involvement. The imaging also showed a prevertebral abscess at the C6 level with posterior subligamentous extension into her spinal canal, causing mild posterior displacement and compression of her cervical spinal cord (Figs. 1, 2). No indication of infarction, hemorrhage, or myelomalacia was found in her cervical spinal cord. Because the infectious diseases consultation service at the referring hospital suspected either TB or a bacterial infection, she was empirically started on anti-TB medications (rifampin, isoniazid, pyrazinamide, and ethambutol) as well as broad-spectrum antibiotics (vancomycin and ceftazidime). While performing an incision and drainage of the prevertebral abscess, otolaryngology at the referring hospital discovered an abscess with a fistulous tract communicating to the C6 vertebral body. AFB smear, Gram stain, and cultures (aerobic/anaerobic, fungal, and TB) were taken intraoperatively. The patient was transferred to our tertiary institution for urgent surgical decompression the same day.

On admission, she was afebrile, with a heart rate of 88/minute, a blood pressure of 132/88 mmHg, a pulse oximetry of 98 %, and a respiratory rate of 14/minute. Laboratory testing found a white blood cell count of 8.9 g/dL, a hemoglobin count of 11.9 g/dL, and an erythrocyte sedimentation rate (ESR) of 94 mm/hour. On examination, she had 5/5 strength in all muscle groups in her upper and lower extremities bilaterally. She had a positive Hoffman sign on the left side and hyperactive bicep and knee reflexes bilaterally. She denied any exposure to wooded/forested areas; however, she did report that she was living in her parent’s unfinished basement where significant construction had taken place including the concrete floor being dug up.

**Fig. 3:**
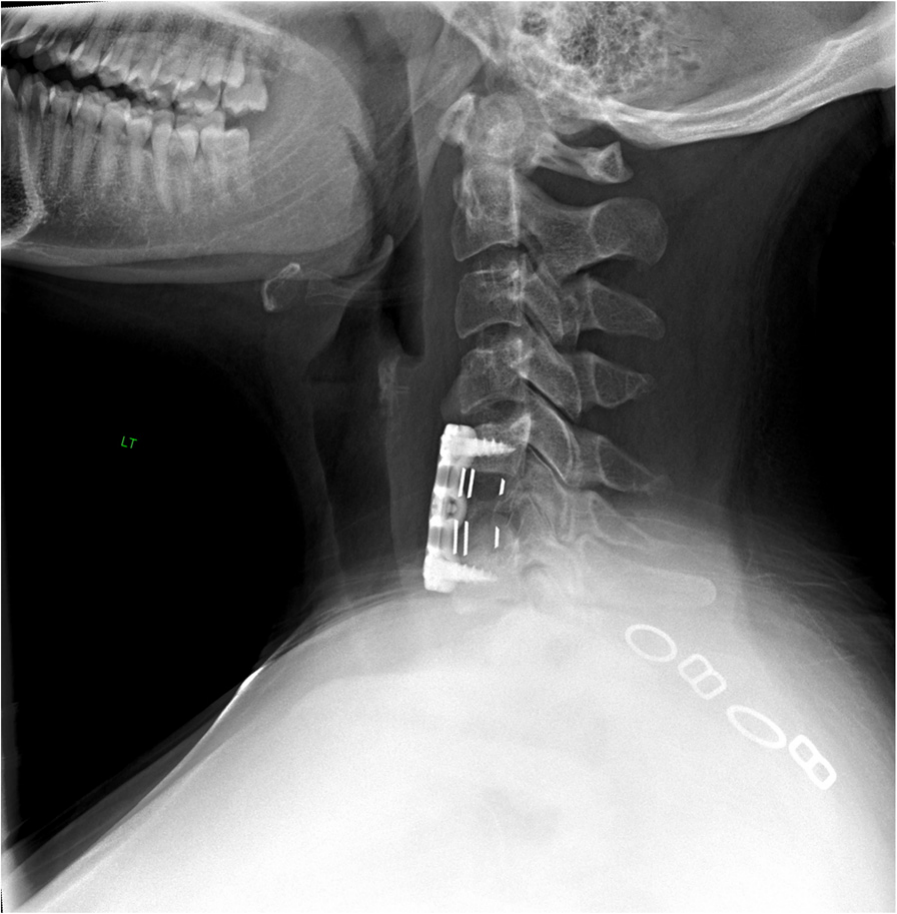
Postoperative lateral cervical spine X-ray showing 18 mm polyetheretherketone cage and 35 mm anterior cervical plate

She underwent emergency C6 ACCF to decompress her spinal cord and restore cervical spine stability. During the procedure, purulent material was expressed from under the prevertebral fascia and was sent for microscopy and aerobic/anaerobic bacterial cultures, fungal cultures, and mycobacterial cultures. Caspar posts were placed in the vertebral bodies of C4 and C7 and distraction was performed followed by exploration and decompression. The C6 vertebral body was found to be completely destroyed; however, the C5 body remained well preserved. After removing the infectious debris, remnants of the vertebral body, and C5 to C6 and C6 to C7 disk material, an 18 mm polyetheretherketone (PEEK) stackable cage (Medtronic, Minneapolis, MN, USA) with demineralized bone matrix (DBM) allograft (Synthes, West Chester, PA, USA) was placed into the C6 space. A 35 mm Atlantis Vision anterior cervical plate (Medtronic, Minneapolis, MN, USA) was secured into the bodies of C5 and C7 with four 12 mm screws (Fig. 3). No complications were encountered during the surgery and the patient had an uneventful postoperative course.

**Fig. 4:**
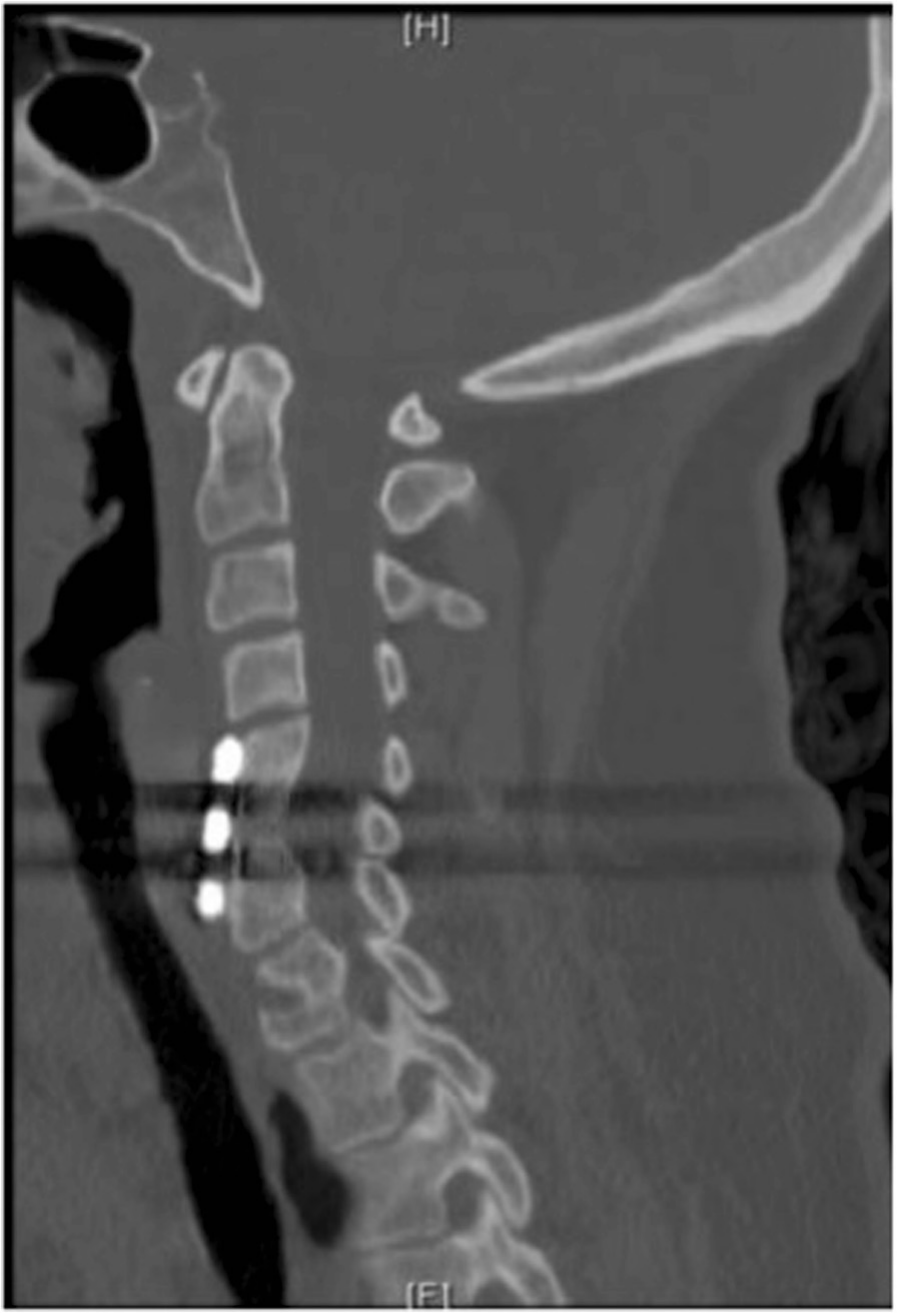
Postoperative computed tomography scan obtained at 17 months showing fusion of C5 to C7

The histopathology was positive for budding yeasts consistent with *Blastomyces* species The anti-TB medications as well as broad-spectrum antibiotics were stopped. The infectious diseases consultation service recommended treating the patient’s osseous blastomycosis and presumed pulmonary blastomycosis with a loading phase of itraconazole 200 mg taken every 8 hours for 5 days followed by a yearlong course of itraconazole 200 mg twice daily. Urine antigen testing for *Blastomyces* species cultures (aerobic/anaerobic), AFB smear, TB QuantiFERON, and human immunodeficiency virus (HIV) testing were all found to be negative. Urine antigen testing for *Histoplasma capsulatum* was reported to be low positive. Fungal cultures obtained at the referring hospital and at our institution were positive for *Blastomyces dermatitidis,* confirming the histopathology findings. The infectious diseases consultation service ensured therapeutic levels were achieved by monitoring the patient’s serum levels during the year-long itraconazole course. A chest CT scan obtained 9 months postoperatively revealed resolution of the mass in the middle lobe of her right lung. At 17 months postoperatively, a cervical spine CT scan was obtained showing C5 to C7 fusion (Fig. 4). At 4 years postoperatively, she had no recurrence and remained pain free.

## Conclusions

*Blastomyces dermatitidis* is endemic to the southern and southeastern states bordering the Mississippi and Ohio River valleys as well as Midwestern states and Canadian provinces that border the Great Lakes [1]. *B. dermatitidis* is endemic in wooded/forested areas around Chicago and the Great Lakes area. The patient lived in an urban environment and denied any recent exposure to wooded/forested areas. Lacking this type of exposure, we suspect that the moist soil exposed during the course of the basement construction, could possibly have been the source of the patient’s exposure. Osseous involvement is the third most common location for *Blastomyces dermatitidis* after lungs and skin, with ribs, long bones, skull, and vertebrae being the most common bones involved [1]. While vertebral osteomyelitis most commonly affects the lower thoracic and lumbar areas of the spine, a few reports of cervical, upper-thoracic, and sacral involvement have been previously reported [5]. We suspect that in our patient, undiagnosed pulmonary blastomycosis seeded the C6 vertebral body when the patient fell and injured her neck.

Fungal infections of the spine are uncommon and relatively difficult to diagnose with an average delay of 3 months [5]. Delays in diagnosis and treatment are also associated with poorer outcomes [5]. Patients with vertebral blastomycosis often clinically present with constitutional symptoms, local bone destruction, and the formation of a contiguous abscess as was seen in our patient; however, these findings are nonspecific and can be seen in patients with TB and other fungal infections [5]. In patients with TB and other fungal infections, bony destruction often begins with the anterior vertebral body and then progresses more posteriorly, resulting in a wedge-shaped compression fracture [5]. We suspect that this pattern was not seen in our patient due to the patient’s late presentation and extensive vertebral body destruction. Other common radiographic findings include vertebral body collapse and disk-space narrowing; vertebral body collapse was seen in our patient (Fig. 1) [5]. In addition, patients frequently develop abscesses that track through the soft tissue with a visible subcutaneous mass [5].

While there are many clinical and radiographic findings, none are pathognomonic for blastomycosis. Definitive diagnosis can only be made with the visualization of the characteristic broad-based budding yeast or growth of fungus in cultures. The fungal smear and culture on initial ultrasound-guided aspiration of neck mass was negative. We believe that the failure to isolate a causative fungal species at that time is most likely due to inadequate sampling, given both were negative [1]. Urine antigen testing has been reported to have sensitivity and specificity of 92.9 % and 79.3 % respectively for all forms of blastomycosis, pulmonary and disseminated [6]. In our patient, the *Blastomyces* urine antigen was negative and the *Histoplasma* urine antigen was low positive. Durkin *et al*. found that high cross-reactivity exists between mycoses because the epitope detected shares a common glycoprotein antigen [6]. This makes the test less valuable for differentiating between fungal species [6]. If urine antigen testing is available at one’s institution, it can help clinicians differentiate fungal infections from TB and guide early treatment until the causative species is definitively identified.

Vertebral blastomycosis has been successfully treated nonoperatively with antifungal medications [1, 2, 7–9]. In patients with moderate to severe disseminated extrapulmonary blastomycosis or with central nervous system (CNS) involvement, the Infectious Diseases Society of America’s clinical guidelines for the management of blastomycosis recommend the use of amphotericin-B lipid formulations until clinical stabilization and then a course of itraconazole [10]. The infectious diseases consultation service recommended using itraconazole in our patient because she lacked CNS involvement. Extended spectrum azoles, such as voriconazole and posaconazole, can also be utilized for treatment [10]. A growing number of case reports have documented successful treatment of patients with blastomycosis using voriconazole and posaconazole [10, 11]. Surgical decompression and stabilization should be reserved for those with neurological deficits, instability, and/or deformity, as was the case with our patient [1, 7, 8, 12, 13].

Preoperatively, external fixation using a halo vest was considered until resolution of the active infection, at which point the patient would undergo definitive fixation. However, we believe that an ACCF with anterior instrumentation is not contraindicated in patients because *B. dermatitidis* lacks glycocalyx biofilm that might hinder eradication [8]. As a result, we conclude that the benefit of performing a single-staged procedure with debridement and definitive fixation outweighs the risks of a multi-staged procedure. The remnant infected C6 vertebral body was considered to be an inappropriate and inadequate source of cancellous autograft bone, thus DBM was chosen as a substitute for fusion purposes. While the C5 vertebra was found to be well preserved intraoperatively, there was still concern that it may have been compromised and be at increased risk for subsidence. The decision to use a PEEK cage was made for its biomechanical properties, namely its elastic modulus. The elastic modulus of PEEK cages matches cortical bone more closely than titanium mesh cages, which may reduce the risk of subsidence [14, 15].

We report the successful treatment of a patient actively infected by *B. dermatitidis,* with concurrent cord compression and cervical spine instability by performing ACCF and treatment with itraconazole for 1 year. ACCF allowed for simultaneous debridement, decompression, and restoration of cervical spine stability. This treatment regimen resulted in the absence of clinical or radiologic evidence of disease at 4 years follow-up.

## Consent

Written informed consent was obtained from the patient for publication of this case report and accompanying images. A copy of the written consent is available for review by the Editor-in-Chief of this journal.
